# An Exergames Program for Adolescents With Type 1 Diabetes: Qualitative Study of Acceptability

**DOI:** 10.2196/65665

**Published:** 2025-05-28

**Authors:** Selene S Mak, Laura M Nally, Juanita Montoya, Rebecca Marrero, Melissa DeJonckheere, Kevin L Joiner, Soohyun Nam, Garrett I Ash

**Affiliations:** 1 Center for the Study of Healthcare Innovation, Implementation and Policy (CSHIIP) VA Greater Los Angeles Healthcare System Los Angeles, CA United States; 2 Department of Pediatrics Yale School of Medicine New Haven, CT United States; 3 Department of Internal Medicine Yale School of Medicine West Haven, CT United States; 4 Department of Health and Movement Sciences Programs Southern Connecticut State University New Haven, CT United States; 5 Department of Psychiatry Yale School of Medicine New Haven, CT United States; 6 Department of Family Medicine University of Michigan Ann Arbor,, MI United States; 7 Department of Health Behavior and Biological Sciences School of Nursing University of Michigan Ann Arbor, MI United States; 8 Yale School of Nursing Orange, CT United States; 9 Department of Biomedical Informatics and Data Science Yale School of Medicine New Haven, CT United States; 10 Center for Pain, Research, Informatics, Medical Comorbidities and Education Center (PRIME) VA Connecticut Healthcare System West Haven, CT United States

**Keywords:** diabetes education, exercise, lifestyle modification, pediatrics, psychosocial-behavioral modification, diabetes mellitus, type 1, adolescent, self-management, exergames, qualitative study, acceptability, physical activity, youths, children, interviews, physical activity intervention, virtual intervention, video game, awareness

## Abstract

**Background:**

Numerous barriers to moderate to vigorous physical activity exist for youths with type 1 diabetes (T1D). The virtual exercise games for youth with T1D (ExerT1D) intervention implement synchronous support of moderate to vigorous physical activity including T1D peers and role models.

**Objective:**

This study aims to understand the acceptability of this intervention to participants.

**Methods:**

We conducted postprogram, semistructured, televideo interviews with participating youths to elicit perspectives on the acceptability of the intervention and experience with the program. Two coders independently reviewed and analyzed each transcript using a coding scheme developed inductively by senior researchers. Discrepancies were resolved by team discussion, and multiple codes were grouped together to produce 4 main thematic areas.

**Results:**

All 15 participants provided interviews (aged 14-19 years; 2 nonbinary, 6 females; median hemoglobin A_1c_ level of 7.8% (IQR 7.4%-11.2%), 5 with a hemoglobin A_1c_ level of ≥10%). Qualitative data revealed four themes: (1) motivation to engage in physical activity (PA)—improving their physical capabilities and stabilizing glucose levels were cited as motivation for PA and challenges of living with T1D were cited as PA barriers; (2) experience with and motivation to manage diabetes while engaging in PA—participants provided details of accommodating the inherent uncertainty or limitations of PA with diabetes and sometimes preparing for PA involved psychological and motivational adjustments while some relayed feelings of avoidance; (3) peer support encouraged engagement with the intervention—participants appreciated the peer aspects of components of ExerT1D and participants’ reflections of the facilitated group experience highlight many benefits of a small-group virtual program; and (4) improvements in PA and diabetes self-management efficacy—all participants credited the program with improving or at least raising awareness of T1D management skills.

**Conclusions:**

Our virtual PA intervention using an active video game and discussion component provided adolescents with T1D the confidence and peer support to engage in PA, improved awareness of diabetes-specific tasks to prepare for exercise, and improved understanding of the effect of PA on glucose levels. Engaging youths with a virtual video game intervention is a viable approach to overcome barriers to PA for adolescents with T1D.

**Trial Registration:**

ClinicalTrials.gov NCT05163912; https://clinicaltrials.gov/ct2/show/NCT05163912

## Introduction

Numerous changes that occur during the adolescent years present unique challenges to youths with type 1 diabetes (T1D). During this time, youths experience multiple psychosocial obstacles, including depression, anxiety, diabetes-specific emotional distress, and diabetes stigma [1]. Furthermore, adolescents have the highest glucose levels of any age group with T1D [2], which put them at greater risk of developing long-term diabetes complications (blindness, kidney failure, and cardiovascular disease). Navigating this transitional time is difficult, underscoring a vital need for interventions to provide psychosocial and educational support for adolescents with T1D.

While physical activity (PA) is well known to improve cardiovascular and mental health [3], it brings unpredictable blood glucose changes. Numerous hormones that affect glucose levels are released in response to different types of physical activity, including catecholamines, growth hormone, cortisol, and glucagon [4]; likewise, insulin sensitivity during exercise can be impacted by stress and overall energy stores [5], and incorporating all of these factors makes diabetes-specific management decisions difficult [6]. The resultant fear of hypoglycemia and diabetes distress also impedes PA [7], as does stigma, especially in group settings due to the visibility of T1D devices during PA, making them feel isolated and exposed [8,9]. Moreover, adolescents report teachers and coaches have limited T1D knowledge [10], and parents may discourage PA [7]. In summary, there are underexplored bidirectional relationships among psychosocial concerns, glycemia, and low PA among teens with T1D. Thus, it is no surprise that adolescents with T1D engage in even less PA than the general adolescent population [11]. Further, few studies have provided exercise self-management education and guided goal-setting for adolescents with T1D [12-17].

A less explored area of equal relevance is the impact of peer support [18]. Peer support involves engaging others with a shared experience and can be emotional (eg, empathy about glucose destabilization from PA), informational (eg, sharing strategies for glucose management), or instrumental (eg, testing glucose together before PA) [19]. Notably, adolescents with T1D see support from peers and peer mentors as important for general well-being [20-22].

Peer interactions among adolescents in the 21st century occur primarily over digital media. Leveraging this affinity, at least a dozen studies have tested digital, mostly asynchronous peer support interventions for adolescents with T1D to discuss diabetes self-management [23]. We sought to build upon them with the virtual exercise games for youth with T1D (ExerT1D) intervention, where participants are focused on performing movements engaging large muscles (ie, exergaming) and experiencing aspects of PA that include not only diabetes management education but also realized PA achievements, in a synchronous virtual room with peers.

Assessing acceptability is an important consideration when designing interventions. Adopting the definition of acceptability from the taxonomy of Proctor et al [18] of implementation outcomes, acceptability is defined as “the perception among implementation stakeholders that a given treatment, service, practice of innovation is agreeable, palatable, or satisfactory.” Studies to assess acceptability enable researchers to identify potential facilitators and barriers to an intervention by eliciting participant feedback. Findings can inform modifications to an existing protocol to further improve the intervention prior to attempting large-scale clinical trial or scaling up the intervention. To understand the acceptability of ExerT1D, we conducted semistructured interviews with all enrollees to complement quantitative evaluations reported elsewhere, in which participant acceptability of this intervention was demonstrated with results from survey data [19]. This paper will focus on the findings from a qualitative evaluation by eliciting participant perspectives on the acceptability of various aspects of the intervention as well as implications for their self-management practices to provide insight into the acceptability of ExerT1D.

## Methods

### Participants and Recruitment

The study was approved by the Yale University institutional review board (2000030105) and registered on ClinicalTrials.gov (NCT05163912). Eligibility for the intervention (see the Intervention Overview section) included youths aged 14-19 years with a T1D diagnosis for at least 6 months, meeting 60 minutes of moderate to vigorous PA (MVPA) fewer than 50% of days, and participants needed to be able to read and speak English. Participants needed to be seen regularly by a clinician specializing in diabetes who could be contacted for safety concerns or insulin dose adjustments. Recruitment occurred at the Yale Children’s Diabetes Center and through an internet posting circulated on Facebook by the nonprofit Children with Diabetes (T1-Today, Inc) which provides education and support to youths with T1D and their families.

### Ethical Considerations

This study was approved by the Institutional Review Board of the Yale Human Investigation Committee (#2000030105). Informed consent or parental permission and assent (younger than 18 years), including the option to be audiotaped for interviews (all participants accepted), were completed prior to any study activities. Participants were required to complete an institutional review board–approved assent form in English, while their parents completed a parental permission form (consent) in English or Spanish. Participants were excluded if they had a medical condition that would limit MVPA participation. Consent was performed in either English or Spanish by our study team. Spanish-speaking families were included as long as the participant met the criteria of reading and speaking English. The research team included a speaker fluent in Spanish (JM).

Each participant was given a unique study identifier to protect their privacy. Interviews were transcribed, the transcript was checked against the recording for transcription errors, identifying information from the interview transcripts was redacted, and then the recording was deleted.

Completion of both glucose safety checks each week (start of Saturday session, start of Wednesday session) was compensated by US $10 for week 1 and then US $5 for consecutive weeks (US $15 for week 2, US $20 for week 3, and so on) with a reset to US $10 if missing a week. There was also US $50 compensation for completing the follow-up visit. The total possible compensation was US $185.

### Intervention Overview

Briefly, the first study of ExerT1D [19] (from December 23, 2021, to July 27, 2022) was a pilot feasibility and acceptability study where 4 cohorts of 3-5 teens with T1D completed the 6-week ExerT1D program, led by a young adult role model with T1D, a T1D clinician, and an exercise physiologist. The intervention included an MVPA video game with a customizable player avatar (Nintendo Ring Fit Adventure), continuous glucose monitoring (CGM) by Dexcom G6 or Freestyle Libre 2, physician-led education regarding managing T1D around MVPA, habitual MVPA goal-setting with a Fitbit Inspire 2 device, and role-playing skits that involve educational points related to T1D management. All activities were done synchronously with the other participants digitally. The curriculum content was iteratively refined after each cohort. The final curriculum content, metrics of feasibility, fidelity, safety, glycemic changes both acutely with exercise and as biweekly summary metrics over time, and exit surveys about acceptability will be reported separately [19]. This paper reports on semistructured exit interviews that accompanied the surveys.

### Interviews

Interviews were conducted by a graduate student (JM) external to the intervention, who was trained in qualitative methodology and interviewing, and mentored by senior members of the research team. The interviewer is female and has a bachelor’s degree while pursuing a master’s degree in exercise physiology. She wrote a field notes summary after each interview and a weekly reflective journal and discussed both with the principal investigator (GIA) on a weekly basis in the first month and biweekly thereafter. A semistructured interview guide [20] was developed by the research team to elicit participant perspectives about the acceptability of the intervention, including their experiences with the intervention components and how these experiences affected their attitudes and perceptions of diabetes self-management practices. Questions also addressed psychosocial experiences with the program and suggested improvements for the program. The first draft of the interview guide was developed collaboratively among the senior investigators (GIA, LMN, and SN) based on questions from previous interventions. This draft was discussed and revised after the first cohort had completed the intervention to incorporate questions about the implementation of the intervention. The interview guide was piloted by the interviewer (JM) in 2 mock interviews with another team member. A senior researcher (GIA) reviewed the recordings of both mock interviews and offered feedback to the interviewer. Recordings of the first 4 participant interviews were subsequently reviewed by senior researchers (GIA and SSM) to iteratively coach the interviewer to improve on open-ended probes. The list of probes was added to the final guide (Multimedia Appendix 1).

Interviews were conducted no more than 7 days after the end of the program using Zoom (Zoom Video Communications), a HIPAA (Health Insurance Portability and Accountability Act)-compliant videoconferencing platform. The interviews were recorded by audio only and transcribed by a professional transcriptionist, and the transcripts were reviewed prior to analysis and updated for accuracy by a study team member.

### Positionality Statement

The interview and coding team reflected on their personal experiences and biases. The team acknowledged their positions of privilege and the fact that some team members live with T1D, which may influence the conducting of interviews and interpreting the data. The multidisciplinary team included a broad range of expertise, including endocrinology, public health, qualitative methodology, and exercise science, as well as social and cultural factors that impact health. This was a collaborative team project that ensured the study was sensitive and appropriate to the context in which it was conducted.

### Data Analysis

Data management and analysis were supported by NVivo 12 (Lumivero). Two senior members of the study team (GIA and SSM) developed a coding scheme [21] based on a literature review for relevant themes and adapted social cognitive theory to guide the analysis. Social cognitive theory predicts that individual health behavior, such as MVPA, is influenced by individual experiences, environmental factors, and the actions of others [22]. The initial coding scheme was piloted with 3 transcripts. Revisions were made after comparing codes, reviewed by another team member (LMN), and discrepancies resolved. The codebook was revised by grouping multiple codes together to produce four main thematic areas: (1) motivation to engage in physical activity, (2) experience with and motivation to manage blood glucose while engaging in physical activity, (3) peer support encouraged engagement with the intervention, and (4) improvements in physical activity self-efficacy and diabetes self-management efficacy. Two members (JM and RM) piloted the revised coding scheme by reviewing 3 other transcripts independently. Codes were compared and discussed, and a codebook was finalized. The coders (JM and RM) used the codebook to code all remaining transcripts independently and met biweekly to resolve discrepancies to maintain rigor in the analytic process. Trustworthiness was ensured by having the same team members involved in the analysis, and the team returned to the interview data to check themes with each participant’s interview and original quotations as needed.

The purpose of the qualitative descriptive analysis is to describe the acceptability and overall impact of the intervention from the perspectives of adolescent participants. A descriptive approach [24] was used to understand participants’ experience with the program, in which there was a heavy reliance on the participants’ words, except when abstracting meaning or intent that fit well-established biomedical or psychosocial aspects of T1D care. The themes are presented with representative quotes and participant descriptors. Adolescents’ ages are described as either “younger” (aged 14-15 years) or “older” (aged 16-19 years) to protect participant anonymity. We applied the COREQ (Consolidated Criteria for Reporting in Qualitative Research) in the reporting of our qualitative study (Multimedia Appendix 2) [25].

## Results

### Demographic Characteristics

A total of 15 adolescents enrolled in the program: median age 15.4 (IQR 14.5-16.4; range 14.1-19.7) years, mixed-sex (2 nonbinary, 6 females, and 7 males), median hemoglobin A_1c_ level of 7.8% (IQR 7.4%-11.2%; range 6.2%-12.5%), average T1D diagnosis duration 8.2 (SD 3.8; range 2.6-14.6) years, and used automated insulin delivery (AID, n=3), a sensor-augmented pump (SAP, n=11), or multiple daily injections (MDIs) on a basal-bolus regimen (n=1). At baseline, they had an average of 1.5 (SD 1.1, range 0-3) days per week with 60 minutes and more MVPA. Baseline PA included school physical education (n=2), formal extracurricular activities (n=3), both (n=1), informal group activities (n=1), or informal individual activities (n=4, of which 1 was exempt from school physical education). During the intervention, they set individual and group goals related to daily steps, sports participation, or playing the active video game on their own time with approximately 40% success [19]. None of the participants experienced diabetic ketoacidosis, severe hypoglycemia, or physical injuries during the study. The protocol was published elsewhere [19]. All 15 adolescents participated in a semistructured exit interview. Interviews lasted between 18 and 48 minutes.

### Interview Themes

#### Overview

**Figure 1:**
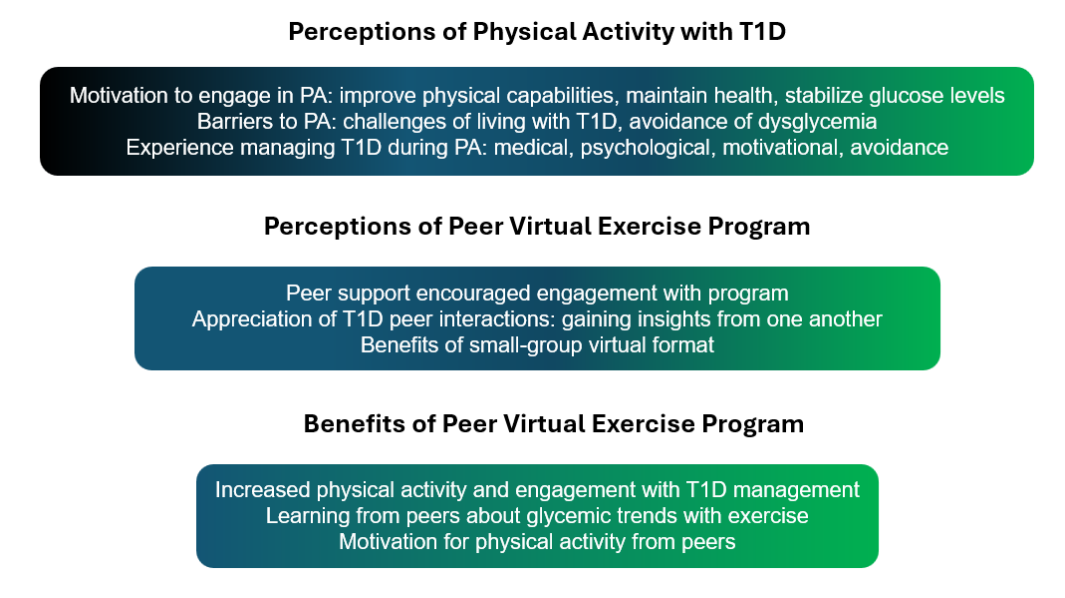
The interview themes related to perceptions of PA with T1D and perceptions of the ExerT1D peer virtual exercise program. PA: physical activity; T1D: type 1 diabetes.

We begin by outlining the participants’ perceptions and experiences with PA and diabetes self-management to offer context for how adolescents with T1D view their biopsychosocial experiences related to the condition. This is followed by participants’ perceptions of and experiences with ExerT1D and how their participation in the program might have affected their views on PA and diabetes self-management (Figure 1).

#### Motivation to Engage in PA

Participants were asked to reflect on their goals, motivation, and barriers to PA to better understand what motivates them to engage in PA. Almost all participants mentioned that they had participated in some form of organized PA before this research study. Activities included running, going to the gym, soccer, volleyball, dance, gymnastics, and flag football. When asked about goals related to PA, many participants shared their desire to improve their current physical capabilities. Specifically, a few of the participants’ main motivation to remain physically active was to maintain good health or to stabilize glucose levels, or both.

I just wanna be, you know, the healthiest I can...the healthiest in terms of like blood sugar especially, and that could be helped a lot by, being physically active, so I think...having good blood sugar is, is what motivates me.

Male, older, MDI

I feel like when I exercise my blood sugars are more stable throughout the day, so exercise usually is a part of my daily routine.

Male, older, MDI

Almost all participants said that an external barrier to being physically active was having other priorities like work, school, extracurricular activities, and other obligations like taking care of younger siblings or house chores. Many of the participants mentioned having unstable glucose levels and other medical conditions (such as asthma) as internal barriers to PA.

Sometimes, if I’m having like a bad blood-sugar day, which isn’t too often, but if do then I’m just like really wiped out, and then I don’t feel comfortable exercising that day, so that’ll stop me from exercising sometimes, or if I’m having like, frequent low episodes throughout the day that’s like a big reason not to, since exercise can make it lower, for me at least.

Male, older, MDI

A few participants mentioned they currently do not make time to exercise and expressed that they wished to devote more time and consistency to exercise.

Further, participants shared how they felt when they engaged in PA. One participant mentioned that this study was “the first time [she] ever truly exercised.” Another participant mentioned that diabetes affected their relationship with PA but “as long as I have, like all the things I need, and make sure I’m on good settings with my pump and all that, then it’s not really that much of a problem.” One participant was adamant about their distaste for PA due to the feelings it invokes.

My whole life, I have hated exercising...[it’s not] even like, the fact that I’m lazy, like I could exercise if I wanted to, but it’s just [the] feeling, that like during it and afterwards I’m like ‘eww, no.’

Nonbinary, younger, SAP

We see that the participants are busy adolescents living full lives filled with family obligations, school, and extracurricular activities. Most participants acknowledged the importance of engaging in PA, but the additional challenges of living with T1D can be a barrier to engaging in PA for some. Several mentioned avoiding PA to prevent hypoglycemia and having to ingest carbohydrates before engaging in PA. Others mentioned following standard recommendations by keeping food with them in case their glucose level got too low. We discuss in more detail how participants manage diabetes in the next section.

#### Experience With and Motivation for Diabetes Management While Engaging in PA

Participants shared personal experiences related to living with T1D and how this influenced their interactions with peers and feelings about themselves. Some avoided discussing diabetes, while others felt managing diabetes was difficult to explain.

I barely even talk about my diabetes to anybody...like I will go months knowing somebody they won’t know I’m diabetic and I’ll avoid it at all costs.

Nonbinary, younger, SAP

[There were] times where I’m like, at dance, or, maybe like in school doing gym and stuff I’ve been low, and it’s, kind of, hard to explain to people why I’ll stop and I’ll have a juice or something.

Female, younger, SAP

Others shared how they had felt abnormal and burdened by diabetes-specific tasks.

I remember like being younger, I was like, ‘Oh my god, I wished I was just like a normal kid that didn’t have to do all this extra stuff.

Nonbinary, younger, SAP

Participants also described how they medically managed diabetes and what they did to stabilize their glucose levels during exercise. Some described using diabetes technology (ie, CGM) to help them track and manage their glucose levels. They also described how they are using diabetes technology, adjusting carbohydrate intake, and using routines to help them manage diabetes.

I’ll usually just have gummies with me and I’ll check my blood sugar before [exercising].

Male, older, SAP

[The CGM] kinda helps regulate [my blood sugar], I feel fine. Like if I do go low, I’m not like scared or anything so, I’m pretty good about exercising.

Male, older, SAP

They also described moments where they experienced low or high glucose levels and actions they took to stabilize glucose levels before engaging in PA.

Depending on...my numbers, I’ll either drink a little juice before...and depending on the exercise I’ll get...a little bit of insulin, but...for exercising, I like [my glucose levels] to be a little higher.

Female, younger, SAP

I think, a couple times, I did, in the morning sessions...when I woke up, I was a little bit low or like going low so I'd set a temp basal to counteract that, but other times I would eat before it so...that would also, stabilize my blood sugar.

Male, older, SAP

One participant explained how she used her CGM to help her figure out diet and insulin adjustments during and after exercise.

I also have a Dexcom that [monitors] my blood sugar so I can give corrections...if I’m...above 70 it’ll give me an alarm saying like ‘hey, you’re 70, eat something.’...So it, tells me when I need to do something about my blood sugar. But, if I’m really high and doing exercise, I might give myself insulin but, it would be,...half of the normal [dose] and, see if it does anything...After the exercise, I might just [give the rest] later, like after it to see if it...is making a big difference.

Male, younger, SAP

While participants expressed some understanding of managing glucose levels during PA, they provided details of having to accommodate the uncertainty or limitations related to diabetes.

Sometimes, during soccer games, I would have to go to the sideline to [treat a low], and I wouldn’t be able to play during that game until...my blood sugar got up, and sometimes it’s annoying or difficult because you can’t play while you have to watch your friends...

Male, younger, AID

Not all days are the same, so, it makes it harder to manage, because you never know what to expect, [you] just have to wake up and see what happens...it’s hard seeing the ups and downs.

Female, older, AID

Sometimes preparing for exercise involves psychological and motivational adjustments. One participant mentioned that if they were experiencing hypoglycemia, it could prevent them from exercising. Another participant talked about pushing through with hyperglycemia.

[If my] blood sugar’s too low, I [wouldn’t] participate really because I don’t want it to go any lower. Or if it’s too high, and I have ketones then I normally don’t do it, but if it’s high and I don’t have ketones I [can], just, [push through]. I’ll become a little bit more sluggish, and it’s, not my best effort...But that’s really all it affects.

Male, younger, SAP

#### Peer Support Encouraged Engagement With the Intervention

One of the main objectives of the group program was to integrate skillful competence (ie, managing T1D around exercise) with relatedness, as per self-determination theory. The components of the program were carefully designed to facilitate and promote interactions between the instructor and participants and between the participants themselves. We asked about the adolescents’ perceptions of the virtual peer experience and how the class format affected their experience.

A little more than half of the participants noted that the instructors of the program were “nice,” “respectful,” and “very supportive.” One participant said.

If the instructors weren’t nice...I may not have stayed with it.

Male, younger, SAP

One participant said the instructor’s demeanor eased his anxiety about meeting new people.

It was just the way they came in with such a high spirit, well the...instructors at least, I know, as I was coming in [you] would probably be a little bit nervous just because you’ve never seen anyone before...

Female, younger, SAP

Another shared that they felt it was easy to communicate with the instructor because knowing the instructor also had T1D.

Pretty comfortable for me to go and tell them things that were happening.

Female, older, AID

Participants also felt the instructors were knowledgeable about living with diabetes and were helpful when glucose-related issues came up. One participant also mentioned her positive experience with the physician.

I knew she had a lot of knowledge in [diabetes], so I, felt like I could trust her and what, what she had to say, in what, what changes she wanted to make.

Female, younger, SAP

It was evident from the interviews that participants appreciated gathering as a group for discussion and gameplay. Although we are unable to compare the experience of adolescents who joined this program and adolescents who play the game by themselves, our participants’ reflections on the group experience highlighted many benefits of having set up the virtual program in a group setting as opposed to providing the game to adolescents to play on their own. Many of the participants expressed that the inclusion of peers around their age with T1D enhanced the acceptability of the program.

I liked that it was other like, teenagers doing it, so I didn’t feel like obviously they were like, older than me, but I didn’t feel like it was, like young young kids that I couldn’t really relate to at all or [it] was like, older people that I was like, “okay, why am I exercising with a bunch of adults?

Nonbinary, younger, SAP

I don’t think I, would’ve enjoyed it as much if it was, just the adults...but, with other kids my age, it was a little more like, relatable and, easier to, talk to.

Male, younger, SAP

I haven’t met many [people with diabetes] my age.

Male, younger, AID

The small-group setting provided a forum for adolescents and instructors to share experiences, provide peer support, and set personal goals. These features are in line with the information-motivation-behavioral skills model, as the program provided information feedback to reinforce motivation for PA. One teen thought the group setting helped him feel more comfortable exercising.

...having someone, there, not, that’s not just me, felt like it, it probably divided a little bit of the attention, which I think is good, because too much attention can be bad, sometimes.

Male, younger, SAP

Another participant explained that group activities helped them cope with social anxiety.

I have like bad social anxiety, so it’s like nice, getting to know people.

Male, older, AID

The peer modeling aspect of the program provided motivation for some participants.

[The other participants gave] me like a sense of progression...it was interesting to see how far they were, compared to me.

Male, younger, SAP

...we would be asked like certain questions every single day, and so we would actually hear like what [the other participants] were saying and how they felt about the whole exercise.

Male, older, SAP

Goal-setting was another important component of the program. By sharing personal goals with each other as well as agreeing on a group goal, the adolescents established intrinsic and extrinsic accountability as motivation to achieve their own goals and the group goal.

I know someone made a goal to run, I think it was run a mile in...six minutes and forty-five seconds...and another one was to get to...twelve thousand steps, I believe, and the other one was to get...A1C down. I felt like that was another one I could’ve done, because I know with the, study, it brought my...A1c down a lot, and I know one other guy did beat his goal.

Female, younger, SAP

In the program you have a group goal, so, our group goal was to get like, ten or fifteen miles a week, and I think that’s one of the things that motivated me because I wanted to get the group goal...so, that motivated me to go outside [and] walk around [with] my sister or, play with the dog, run around more with the dog, and things like that.

Female, older, AID

Participants expressed an interest in peer responses to living with diabetes and PA. In an effort to normalize the practice of regularly checking and sharing glucose levels, participants were asked to share their glucose levels before and after the game. There were mixed feelings about having to share their glucose levels in the group.

I literally avoid that, at, if I can avoid it, I will. If there’s something else I could do than other show my numbers, I will do that, because I, that’s something very personal to me, like, like for the other kids I feel like it was so much easier,...it just felt so much more, it felt, effortless, it looked effortless for the other kids.

Nonbinary, younger, SAP

I think this game was fine to help the barrier like showing people my blood sugars, I never ever ever do that, that’s something I never do, I hate people seeing my numbers, I hate people seeing when I check my blood sugar seeing my sensors or anything...it makes me so uncomfortable.

Nonbinary, younger, SAP

They not only found it beneficial to learn exercise skills but also learn about how others’ glucose levels responded to exercise and how others managed it.

I thought it’d be fun and I thought it’d be like a different way to do exercise and it would kinda distract me from the fact that it’s exercise, and then...it would be super helpful to see how like diabetic people react, like how their bodies react to exercising...

Nonbinary, younger, SAP

[The blood sugar is] like really usually like a huge issue for me, so I kinda got to learn even just from what other people’s goals were like, I got to be able to like, learn more tips and tricks for myself and for my diabetes.

Female, younger, SAP

Several participants indicated an interest in building community:

I wanted to meet more kids my age [with type 1 diabetes] so I wasn’t really sure how to do that.

Female, older, SAP

Having all participants on Zoom during and after the game allowed flexibility in making connections with other participants. Some enjoyed being able to interact through Zoom during the game, facilitated by the Zoom-Ring Fit hybrid delivery (the Ring Fit game itself is not networked).

[the program was] super cool, having, a, team type of thing, and I feel like you would also be able to get closer with the other person.

Female, younger, SAP

Others found the postgame virtual discussions valuable.

When we were actually in the game we didn’t really interact that much, just because we were focused on our own game, but afterwards, you could like hear everyone’s blood sugars, and how they came down I guess, and you could see everyone else’s goals and, [who had beat] their goals and how, how they got to, beating that goal.

Female, younger, SAP

Some mentioned they did not have much interaction in the program and outside of it because either they chose not to or the platform for the game made it difficult for them to interact with one another.

It was nice. I feel like we didn’t interact as much as we could have...but, for the few interactions we did have, it was nice. Again, everybody was really nice and, sweet and kind.

Female, younger, SAP

#### Improvements in PA Self-Efficacy and Diabetes Self-Management Efficacy

All the participants credited the program with improving or raising awareness of T1D management skills. As a result of the program, participants were more conscious of checking and testing their glucose levels so they could adjust therapy plans and mitigate hypoglycemia and hyperglycemia. They reported having developed pre-exercise planning skills for T1D, such as monitoring their glucose levels more actively and having snacks nearby while exercising as a result of the program. Several participants relayed improved hemoglobin A_1c_ and more success with stabilizing their glucose levels before and after exercising. A few noted that exercising is beneficial for their health since it keeps their glucose levels stable. Their comments demonstrated improved self-efficacy.

I’m trying to be more aware of it now, and, like, consciously checking...

Female, younger, SAP

I know what my blood sugar is, just, being more on top of it.

Female, younger, SAP

I noticed that [in] the program I was testing my blood sugars a lot more, or looking at the Dexcom a lot more, [just] to see what was happening.

Female, younger, SAP

...keeping myself in check because they would ask, what your blood sugar was every morning, [and I thought], “Well I should do something about this [on my own].

Female, younger, SAP

[I now can] exercise for a long period of time, and not be like too worried about my blood sugar.

Male, older, SAP

Even for someone who felt knowledgeable about managing her own diabetes, this participant acknowledged the benefits of participating in a group.

I’ve been doing that for a long time, it just gave me some insight onto, how other people do it, so I can compare what I’m doing with other people.

Male, younger, AID

Participants had high praise for the overall program structure. As previously discussed, participants enjoyed the small-group format with other adolescents living with T1D and developed a rapport with instructors who also had diabetes. Having the game component as well as a discussion or class component gave participants different ways to connect with each other and practice the skills modeled in the program.

Participants were also asked to reflect on their experience with the game. They appreciated learning new exercises through playing the game, as the game’s feedback helped participants know if they were doing the exercise correctly. Some participants credited the game for helping them improve their endurance. One person mentioned the ability to set the intensity level of the exercises allowed participants to match the intensity to how they were feeling. The storyline of the game allowed them to take their mind off the fact that they were exercising; because they liked the competition, it was more enjoyable. They were focused on beating the game rather than performing the exercises.

I don’t really like [exercising], but...because there was a game and everything that just kind of, took my mind away from the fact that it is exercise.

Nonbinary, younger, SAP

One participant said that their favorite part of the program was performing the exercises because of how fun they were. The participants enjoyed the different types of exercises and the feature that allows player to unlock more levels. While the game caused one participant to feel tired because of the intensity level, they expressed excitement about how the game helped them increase their fitness levels.

Some participants talked about how much they enjoy exercising more because of the program. For example, the program was “fun,” and it motivated them to exercise more by having a more consistent workout structure.

It [gave me] a bit, more of a schedule for doing exercises, and, diabetes, I think [you need a bit] more discipline.

Male, younger, SAP

The program gave them the confidence to safely engage in PA and gain a better understanding of the effect of PA on blood glucose levels.

[I was] able to like track my blood sugar and understand the effects of being low or being high.

Male, older, SAP

...that was really helpful with the [Ring]Fit, I kind of got a better understanding of like how far I can push myself without having to...eat a snack.

Male, older, MDI

It helped me like realize that...I can exercise for a long period of time, and not be...too worried about my blood sugar, because I realize that...my blood sugar might even be stable if I eat before [exercise] so it was nice to see that...it made me realize that I can do these more...vigorous exercises and not be that worried about my blood sugar.

Male, older, SAP

## Discussion

### Principal Findings

Through the ExerT1D intervention, we present a novel approach to promote PA while providing psychosocial support for adolescents with T1D utilizing both exergaming and virtual reality. The positive participant responses to the program demonstrated the acceptability of hosting a fully virtual program, as participants enjoyed the small-group format and appreciated the convenience of meeting digitally from their homes. Highly rated components of the intervention included the instructors with lived T1D experience and perceived group cohesion. Using exergames and virtual reality, we provided a fun and interactive environment for participants to interact and engage in PA in our study. Pairing a video game or virtual reality with group activities in a virtual setting proved to be a viable approach to addressing some of the psychosocial challenges that adolescents with T1D experience.

Participants attributed the ExerT1D program to enhancing their diabetes knowledge and management skills, both overall and during PA. The program gave them the confidence to safely engage in PA, and they gained a better understanding of the impact of PA on glucose levels. The combination of increased self-efficacy and peer support can create a positive feedback loop; as youths create supportive peer networks, these supportive relationships can further enhance their confidence in managing diabetes effectively. Other key benefits of the program were the structured nature of the sessions that started by addressing diabetes management prior to PA, as well as real-time feedback about how to manage diabetes during PA. Further, after PA sessions, participants had the opportunity to reflect on how their glucose levels changed during activity and if their diabetes management strategies were successful.

Additionally, adolescents reported that interacting with peers who also had T1D was a valuable aspect of the program. As T1D is a rare condition in childhood, many adolescents with this condition may feel isolated because they do not have the opportunity to engage with others who share similar experiences. The mental burden and constant nature of T1D management can be cumbersome and lead to psychological distress in youth [26]. Participants in this study relayed the complex nature of managing T1D around PA, including the need to consume additional carbohydrates around times of PA, the unpredictable nature of glucose changes, and T1D-specific limitations that arise with the condition. The opportunity to engage with fellow participants and instructors with T1D allowed them to learn from each other and build a sense of community. Sustained peer support programs have also been linked with improved glycemic outcomes [27]; thus, peer support can play a key role in providing motivation and encouragement for managing T1D.

Unlike programs that prescribe PA regimens which are completed independently, our program addresses psychosocial concerns of PA for adolescents by conducting the program in a small-group setting led by peer mentors with T1D rather than automated instructors, which reinforces peer support [28,29]. The setting also promoted diabetes self-management practices, including regular glucose checks before, during, and after PA, and used group discussions by fostering a “safe space” for discussing diabetes challenges. Active video games most effectively increase PA, PA self-efficacy, and PA motivation when focusing on cooperative (ie, cumulative group) scoring [30,31]. In addition, playing through a self-representational figure (ie, avatar) fulfills drivers of motivation for behavior change stipulated by self-determination theory [32].

### Strengths and Limitations

As this was a pilot study, interviews were conducted to evaluate and refine the intervention. We are therefore unable to draw conclusions or propose theories about the effectiveness of the program. Participants were required to own technology to participate, including television, phone, and internet access, which may be a barrier for those with limited financial resources. Participant responses may have been impacted by biases common to many qualitative studies. First, to mitigate social desirability bias, the interviews were conducted by a team member who was not directly involved with any aspect of the program, thus creating a sense of separation from the research team. Second, for recall bias, we scheduled the interviews within 7 days of the completion of the program and provided additional cues to remind participants of specific sessions if appropriate. Third, we also had the same person conduct the interviews to minimize variation in conducting interviews. Fourth, the principal investigator listened to each recording after the interview and debriefed with the interviewer as another quality check. With qualitative studies, although a researcher’s biases may affect the analytic process, we coded the data independently and resolved discrepancies by group discussion to maintain rigor in the analytic process.

### Comparison With Prior Work

Findings from this study further reinforce results from our previous intervention (Bright 1 Bodies), an in-person group PA program for adolescents with T1D [33]. The 18 adolescents in Bright 1 Bodies had similar racial and ethnic diversity and low baseline PA levels to our study. Consistent with comments from this study’s participants, Bright 1 Bodies participants similarly reported experiencing relatedness by meeting others with T1D (in many cases for the first time), learning more about T1D, improving T1D management and experience, and having young adult instructors with T1D as role models. They also reported that the intervention led them to gain and reinforce autonomy and competence in safe, enjoyable PA [34]. In addition, findings from this study suggest it could be beneficial to leverage the clustering of positive health behaviors by initially focusing on the ones that are least stigmatizing (ie, multiple health behavior changes) [35,36]. Structuring the program to be delivered by an instructor living with T1D, in addition to interacting with other participants with T1D, was a key to the success of the intervention. Virtual peer support MVPA interventions may be more effective, acceptable, and implementable than existing options for adolescents with T1D.

Adolescence is a period of development where youths are more vulnerable to psychosocial challenges relevant to MVPA, including avoidant coping behaviors (eg, committing leisure time to sedentary activities) [37] and low self-efficacy (eg, lack of belief that MVPA can be safe and fun) [38]. This intervention is the first program using virtual digital tools to address the bidirectional relationships among psychosocial concerns, glycemic control, and low PA among adolescents with T1D. Prior studies have either focused separately on PA promotion, diabetes management, or psychosocial aspects of T1D. A small number of studies have explored the role of digital tools for adolescents with T1D, with encouraging signals that digital tools may be effective in improving self-efficacy, but evidence for other outcomes is, at best, mixed [23]. Our study was able to shed light on multiple outcomes since this program explored the role of digital support for adolescents with T1D in both PA promotion and psychosocial concerns. In this study, adolescents reported less avoidant coping and greater self-efficacy around MVPA. They also shared that the glucose checks before PA provided safety, comfort, confidence, a sense of normalcy, and awareness of glucose changes during and after PA.

### Future Directions

Findings from this study suggest that targeting T1D self-management skills, particularly in the context of PA, may offer valuable insights for research. The study also underscores the potential benefits of T1D peer support. Given the limited opportunities that adolescents with T1D have to connect with others with shared experiences, peer engagement creates an opportunity for shared learning and social support. Future programs could incorporate more structured opportunities for virtual peer interactions, which are more feasible to implement broadly. While further research is needed to test the effectiveness of such interventions, our study highlights the importance of exploring approaches that integrate practical education, real-time feedback, and peer support to improve self-management of T1D in adolescents.

### Conclusions

Adolescents with T1D experience barriers to engaging in PA. We provided an intervention focused on promoting PA and addressing psychosocial aspects of participating in PA for adolescents with T1D in a virtual setting using digital tools (ie, videoconferencing platform and parallel video gaming) and biosensors (Fitbit and CGM). The intervention provided adolescents with T1D with the confidence and peer support to engage in PA, reinforced by diabetes self-management skill development, and facilitated a greater understanding of the effect of PA on glucose levels. Participant feedback indicated the feasibility and high acceptability of this program, providing invaluable insight into the refinement of this approach for future trials.

## Appendix

Multimedia Appendix 1 Interview guide.

Multimedia Appendix 2 COREQ (Consolidated Criteria for Reporting in Qualitative Research) checklist.

## Abbreviations

AID: automated insulin delivery
CGM: continuous glucose monitoring
COREQ: Consolidated Criteria for Reporting in Qualitative Research
ExerT1D: virtual exercise games for youth with type 1 diabetes intervention
HIPAA: Health Insurance Portability and Accountability Act
MDI: multiple daily injection
MVPA: moderate to vigorous physical activity
PA: physical activity
SAP: sensor-augmented pump
T1D: type 1 diabetes
