# Supplementary material for: An Exergames Program for Adolescents With Type 1 Diabetes: Qualitative Study of Acceptability
Source: JMIR Diabetes. 2025 May 28;10:e65665. doi: 10.2196/65665 (PMC12159555; doi:10.2196/65665)
Supplement: Multimedia Appendix 1 [file diabetes_v10i1e65665_app1.docx]

Multimedia Appendix 2: Interview Guide

**A Virtual Home Intervention to Promote Physical Activity Among Youth with Type 1 Diabetes**

**Virtual physical activity study**

**Good morning/afternoon/evening, my name is Juanita and I am a graduate student at Southern. Thank you for speaking with me today. I would like to talk to you about the research study and I would like to hear your thoughts on the program and your experiences**. This study is enrolling at least 15-20 participants and the responses will all be analyzed together. **This information will help understand the benefit and effect of physical activity on type 1 diabetes and it will help develop physical activity programs that benefit other teens with type 1 diabetes.** **I will record this meeting to more accurately capture what you have to say [if applicable].**

**We will be conducting today’s interview in English; however, I also speak Spanish so if there’s any responses you would prefer to give in Spanish, please feel free to do so.

**[*TURN ON RECORDER NOW*]**

**Today is [date] and I’ve just started the recording. You’ve consented to participating and I’d like to get confirmation of that on the audio recording please.**

• **Do you agree to participate in this interview?**

- **Do you agree to being audio-recorded?**

**TOPICS**

**Unstructured questions:**

- **What is your relationship with physical activity like?**
- **When are you the most active?**
- **What are some of your favorite things to do to say active?**

Question 1: Can you tell me about why you decided to join the research study?

1. a. What was your first reaction to the first group Saturday session?

2. Question 2 – What did you like about the program?

a. What was your favorite part of the program?

b. How often would you want to do the different parts of the program?

3. Question 3 – What were some challenges with the program?

a. What did you like the least about the program?

b. *So, what would you change about this program?*

c. ***don’t give any examples***

4. Question 4- What were the barriers to playing the game during your free time?

a. Could using a different game help fix these barriers?

b. Follow up question: What are some examples of games?

5. Question 5 - Can you tell me how you manage your diabetes? For starters, do you use an insulin pump or injections.

1. What changes do you make to your diabetes management before, during, and after exercise?

6. Question 6 - What are some of your personal physical activity goals?

Y: What helps you achieve those personal physical activity goals?

N: What motivates you to be physically active?

c. Did the program do anything to help you achieve them?

Follow-up question: Can you think back to a time in your life where you achieved your physical activity goals?

7. Question 7 - What prevents you from being physically active?

a. Follow-up question: Did the program address any of these barriers?

b. Can you give me an example of how the program addresses any of the barriers?

8. Question 8 - If you could organize the program and money was no issue, what would it look like?

a. If you could create your own program, what would it be like?

9. Question 9 - Were there any barriers to talking with other teens during the Wednesday night discussions? What about talking with instructors?

1. Were there any barriers to talking with other teens during the Saturday exercise sessions? What about talking with instructors?

b. Follow up question: could these barriers be overcome if we used a different game?

c. What are some examples of games?

d. What mode would you prefer for these sessions (online group, online individually, in-person group, in-person individually)

e. How would you feel about talking with the other teens in a networked game instead of over Zoom? For example, if your friend was having trouble defeating all the monsters, they could call you over to join the battle and help.

**Before we end our interview, do you have any final thoughts or suggestions for us?**

**Thank you for your time. We appreciate the answers you have given us.**

Notes: (follow up, probe)

- Can you tell me more about that? How did that whole conversation unfold?
- what are your thoughts about why you decided to join the program.
- Did you have any favorite discussions? Or anything that you wanted to talk about but you didn’t get a chance to?
- So, you would like the program to just keep going?
- What are some other times you’ve experienced……….. How did this compare to those situations

| **Template for Interview Notes** | |
| --- | --- |
| The overall demeanor of the participants – for example, describe if the participants were sad, uninterested, forthcoming, timid, relaxed, defensive, etc. |  |
| Any interruptions that occurred during the IDI |  |
| Cultural factors mentioned that would be important for others outside the local context to understand |  |
| Questions that were difficult for the participants to understand and suggest modifications |  |
| Issues and/or participant questions that need to be followed-up. |  |
| Any information that contradicts with what you have learned in other interviews. |  |
| Themes or patterns that are emerging [e.g. information that you are hearing over and over again from IDI participants]. |  |
| Additional general notes |  |
